# Response to: Comment on “Acetazolamide Intoxication in an Elderly Patient with Diabetes and Chronic Renal Failure after Cataract Surgery”

**DOI:** 10.1155/2021/9892830

**Published:** 2021-12-29

**Authors:** Juliana Maria Kerber, Juliana Dias de Mello, Karolinny Borinelli de Aquino Moura, Gustavo Cardoso da Silva, Iuri Christmann Wawrzeniak, Tatiana Helena Rech

**Affiliations:** ^1^School of Medicine, Universidade Federal do Rio Grande do Sul, Porto Alegre, RS, Brazil; ^2^Intensive Care Unit, Hospital de Clínicas de Porto Alegre, Porto Alegre, RS, Brazil; ^3^Department of Neurology, Hospital de Clínicas de Porto Alegre, Porto Alegre, RS, Brazil; ^4^Graduate Program in Medical Sciences: Endocrinology, Universidade Federal do Rio Grande do Sul, Porto Alegre, RS, Brazil

We read with great interest the comments from Dr. Schwenk [1] regarding our case report entitled “Acetazolamide Intoxication in an Elderly Patient with Diabetes and Chronic Renal Failure after Cataract Surgery” [2] published in this journal. In the letter, the author questions the helpfulness of renal replacement therapy (RRT) in clearing acetazolamide toxicity levels.

In fact, we could not provide evidence regarding acetazolamide serum levels, as we did not perform acetazolamide quantifications. However, the patient was critically ill, with coma and life-threatening metabolic acidosis refractory to medical treatment, which determined the urgent need for RRT. Besides, RRT was used to treat the progressive neurological decline probably related to the persistent high levels of acetazolamide, as the drug had been withdrawn in the emergency department 48 hours before starting RRT.

We believe that the patient benefited from RRT because of the rapid improvement of neurological performance following therapy, with complete recovery of mental status after two sessions of hemodialysis, as expected for an overdose with a medication that is easily dialyzable [3, 4]. Conversely, uremia symptoms usually take longer to resolve [5]. However, we agree with Dr. Schwenk that this is merely a hypothesis, as acetazolamide serum levels were not quantified.
